# Pathogen Detection via Quantitative PCR in Milk of Healthy Cows Collected Using Different Sampling Protocols

**DOI:** 10.3390/pathogens12070935

**Published:** 2023-07-13

**Authors:** Silvia Magro, Elena Visentin, Elena Chiarin, Filippo Cendron, Mauro Penasa, Angela Costa, Martino Cassandro, Massimo De Marchi

**Affiliations:** 1 Department of Agronomy, Food, Natural Resources, Animals and Environment, University of Padova, 35020 Legnaro, Italy; elena.visentin.10@phd.unipd.it (E.V.); elena.chiarin@unipd.it (E.C.); filippo.cendron@unipd.it (F.C.); mauro.penasa@unipd.it (M.P.); martino.cassandro@unipd.it (M.C.); massimo.demarchi@unipd.it (M.D.M.); 2 Department of Veterinary Medical Sciences, University of Bologna, 40064 Ozzano dell’Emilia, Italy; angela.costa2@unibo.it; 3 Associazione Nazionale Allevatori della Razza Frisona, Bruna e Jersey Italiana, 26100 Cremona, Italy

**Keywords:** mastitis, udder health, qPCR, selective dry cow therapy

## Abstract

In this study we evaluated the prevalence of pathogens detected via quantitative PCR (qPCR) in milk from apparently healthy cows to identify the most common etiological agents present in Italian dairy farms. Milk samples were collected using a sterile protocol at quarter-level (3239 samples, 822 cows) and a conventional protocol at udder level as composite milk from the functional quarters of each cow (5464 samples, 5464 cows). The qPCR commercial kit detected *Mycoplasma bovis*, *Mycoplasma* spp., *Staphylococcus aureus*, coagulase-negative staphylococci (CNS), *Streptococcus agalactiae*, *Streptococcus dysgalactiae*, *Streptococcus uberis*, *Prototheca* spp., *Escherichia coli*, *Klebsiella* spp., *Enterococcus* spp. and *Lactococcus lactis* ssp. *lactis* as well as DNA from the penicillin resistance β-lactamase gene from staphylococci. The prevalence of specific DNA was calculated based on its presence or absence in the samples, factoring in both the sampling protocols and herds. Regardless of the sampling protocol used, the most frequently detected pathogens were CNS (26.6% in sterile and 13.9% in conventional protocol) and *Streptococcus uberis* (9.6% and 16.5%, respectively). These results underscore the necessity for pathogen-specific interventions at the farm level to enhance the udder health of dairy cows via management recommendations.

## 1. Introduction

The presence of pathogens in the mammary gland causes the onset of intramammary infection in dairy cows, which can progress to clinical or subclinical mastitis [1]. In order to reduce costs for veterinary interventions and treatments and limit antimicrobial use, a timely identification of intramammary infection coupled with specific pathogen detection is essential to supporting farmers’ decision-making and move towards a targeted antimicrobial use. For example, at dry-off, European farmers are required to refrain from blanket therapy and perform treatment only on cows at major risk. In fact, the use of antimicrobials in dairy herds for preventive purposes such as the blanket dry cow therapy has been banned in the EU since January 2022 (EU Regulation 2019/6) due to concerns related to antimicrobial resistance in animals and, above all, in humans. The Regulation 2019/6 represents the new veterinary regulation and aims to define guidelines to monitor antimicrobials use and market within Europe. Identification of pathogens and accurate selection of cows—or, even better, quarters—to be treated before the dry-off translate into a smarter and more efficient medicaments management at the farm level [2].

Mastitis-causing pathogens can be classified into contagious, environmental, and opportunistic based on their main reservoirs and transmission mode [3,4]. The main substrate of the contagious pathogens is represented by infected tissues, e.g., the udder. In this category transmission among cows primarily occurs through vectors such as the milking machine or human hands. Environmental pathogens, on the other hand, originate from the surroundings and primarily contaminate the teats of cows outside the milking parlor. Environmental pathogens causing mastitis are particularly present in farms with suboptimal hygienic conditions. Finally, there are the opportunistic pathogens, which normally reside on the cow’s skin and cause clinical or subclinical inflammation only when certain conditions favor their ascendant colonization from the mammary gland ducts [5].

Bacterial culture has been considered the gold standard when identifying pathogens in biological media, including milk [6]. However, more advanced molecular techniques have been recently developed for faster and more specific identification of bacterial species in milk [7], such as *Mycoplasma* spp. [8]. Quantitative PCR (qPCR) identifies the DNA from pathogens commonly associated with mastitis and offers several advantages: (i) rapid detection of mastitis-causing pathogens (approximately 3 h), including pathogens that cannot be grown using conventional culturing techniques [9]; (ii) objective reporting of the DNA found in the milk sample; and (iii) the potential to detect non-living pathogens [10]. In fact, qPCR can also be used in milk samples treated with conventional preservatives such as Bronopol (2-bromo-2-nitropropan-1,3-diol), which has a bactericidal activity. As compared to the bacterial culture, qPCR significantly reduces time of analysis and allows for the simultaneous processing of a large number of milk samples for a variety of pathogens. According to Nyman et al. [11], qPCR may potentially be applied to the composite/pooled samples collected during dairy herd improvement (DHI) tests. This could be useful for mastering selective dry cow therapies and identifying harmful and contagious mastitis-causing agents within each farm [11]. When milk samples are obtained from milk meters, there can be a carry-over of microorganisms’ DNA from one sample to the subsequent ones, i.e. from a cow to the following one. This has been demonstrated for *S. agalactiae* and *S. aureus* [12,13].

In Italy, DHI is carried out according to the standards of the International Committee for Animal Recording [14,15] to ensure consistency and comparability of data within and across countries. Milk test-day records include herd data (e.g., animal ID, milk yield, days in milk, parity) as well as milk composition descriptors as determined by the official laboratories. By mean of the somatic cell count (SCC, cells/mL), farmers can monitor the udder health status of their cows. In recent years, thanks to modern commercial benchtop devices, it has been possible to record additional information about udder health. The the differential SCC (DSCC) in fact has been introduced in some Italian DHI milk laboratories [16]. The flow cytometry satisfactorily distinguishes polymorphonuclear neutrophils and lymphocytes from macrophages in cow milk [16], apparently allowing for a better identification of cows with or without acute, subclinical, and chronic inflammation. Nevertheless, SCC as well as DSCC can vary according to the pathogens responsible for the inflammation and nowadays information on specific pathogens present in the milk is not provided by the standard DHI procedure. 

The present study aimed at evaluating the prevalence of DNA from different pathogens detected via qPCR in Italian farms using milk samples collected under different conditions: quarter vs. composite milk and sterile vs. non-sterile sampling protocol.

## 2. Materials and Methods

### 2.1. Rationale of the Study

This study is part of the research project DOC-AR 2021 (“Dry-Off Cow and Antibiotic Reduction”) of the Breeders Association of Veneto Region (ARAV, Vicenza, Italy). The overall aim of the project was to propose new strategies for the monitoring of mastitis and the reduction of antimicrobial use in the dry period. In total, 12,759 milk samples were collected between June and December 2021 from 6821 cows in 186 commercial herds. Farms were all managed under intensive conditions and were distributed within different provinces of the Veneto region. Cows under veterinary treatment or with signs of mastitis on the day of sampling were excluded from the study. These, in fact, are not typically tested during DHI. Therefore, in the present study, only cows free of clinical mastitis, hereby defined as “healthy”, were considered. It cannot be excluded that subclinical mastitis was present in any of the cows sampled.

### 2.2. Samples Collection

**Figure 1:**
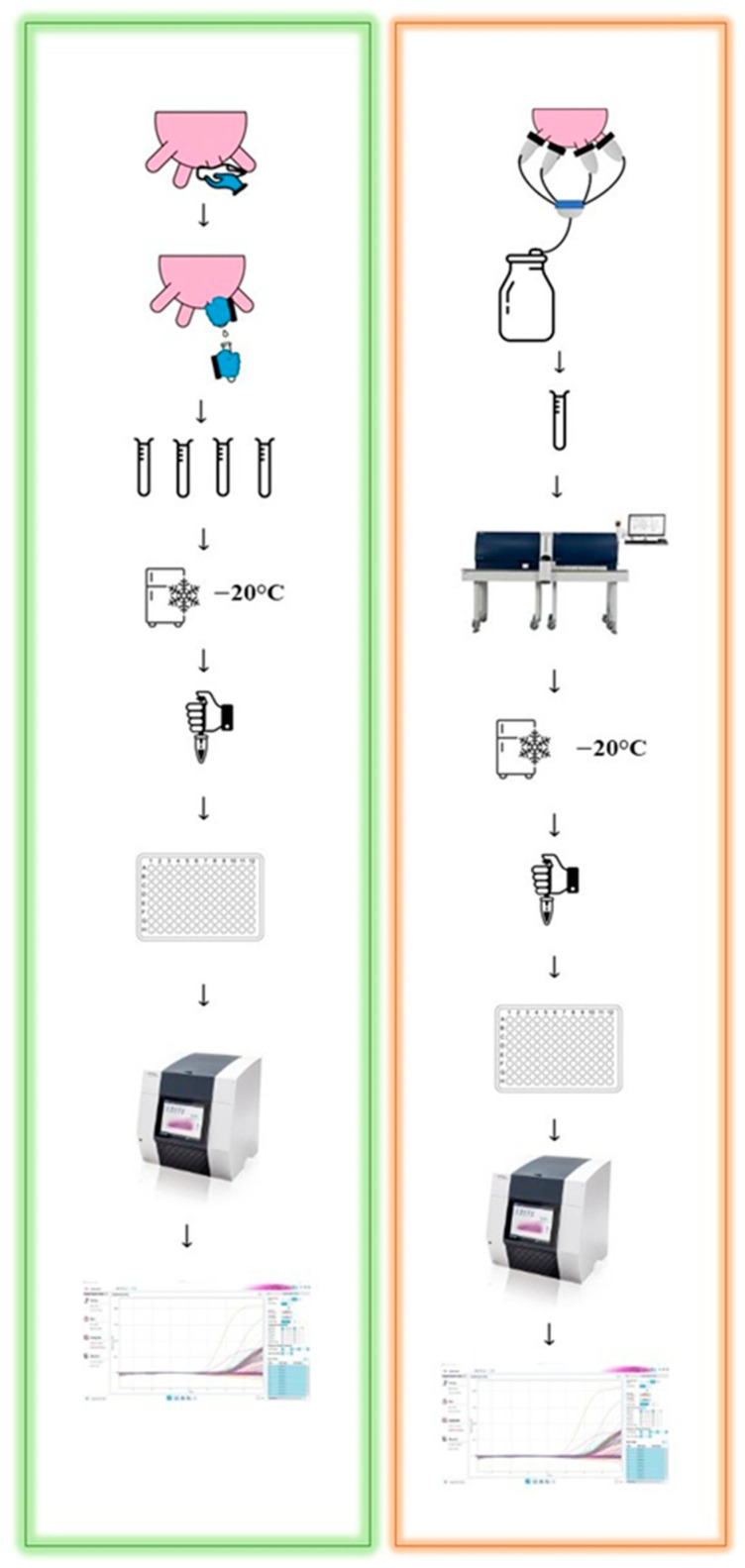
Overview of sterile (**left-hand side**) and dairy herd improvement (DHI; **right-hand side**) procedures for collection of milk samples for qPCR.

To compare the two sampling procedures, milk was collected at both (Figure 1):

- quarter level—under sterile conditions (STER): 9 herds, 3288 quarter milk samples, 822 cows;
- cow level—during DHI milk testing (DHI) [14,15]: 177 herds, 9471 composite milk samples, 5999 cows.

In both sampling procedures, Holstein Friesian, Simmental, Rendena, and Jersey breeds were represented. The 9 STER farms were visited only once and all the lactating cows present were sampled, as none were under any treatment. In the DHI set, each herd was visited more than once during the experimental period. In both cases, sampling procedures were carried out on the day of the DHI test in the presence of ARAV and University of Padova personnel, and milk samples from cows at different lactation stages, parities, and calving season were collected. 

In the STER protocol (Figure 1), each quarter was sampled after a normal pre-milking routine, which consisted of cleaning teats and their orifices with wet disposable towels (Kerbl, Buchbach, DE; 1 per teat). After manual forestripping, approximately 10 mL of milk from each quarter was aseptically collected in sterile tubes containing Bronopol for qPCR. After this step, milking took place in normal (non-aseptic) conditions with the automatic reservation of 50 mL of composite milk for the official analysis. The aliquots containing the quarter milk were stored (−20 °C) at the ARAV milk laboratory until qPCR testing was conducted, whereas the composite milk was refrigerated (4 °C) and processed with the CombiFoss^TM^ 7 analyzer (Foss Electric A/S, Hillerød, Denmark) to assess fat, protein, and lactose contents; SCC; and DSCC. For the DHI (Figure 1), composite milk samples were collected as usual by trained ARAV technicians in conventional non-aseptic conditions [13,14], and the contents of fat, protein, and lactose; SCC; and DSCC were determined as described above. As more farms were visited in a day, milk samples from different herds were often analyzed on the same day in the ARAV milk laboratory. Following analysis with the CombiFoss^TM^ 7, the remaining volume of milk from each sample from the DHI protocol was stored at −20 °C until qPCR testing was conducted, as previously described by Timonen et al. [8].

### 2.3. qPCR

The commercial quantitative “Mastitis 4BDF” (DNA Diagnostic A/S, Risskov, DK) kit already used in previous studies [8,17,18] was purchased to isolate the DNA from *Mycoplasma bovis* (*M. bovis*), *Staphylococcus aureus* (*S. aureus*), coagulase-negative staphylococci (CNS), the penicillin resistance β-lactamase gene from staphylococci (β-lactamase gene; *blaZ*), *Streptococcus agalactiae* (*S. agalactiae*), *Streptococcus dysgalactiae* (*S. dysgalactiae*), *Streptococcus uberis* (*S. uberis*), *Mycoplasma* spp., *Prototheca* spp., *Escherichia coli* (*E. coli*), *Klebsiella* spp. (*K. pneumonie*, *K. oxytoca*, and *K. variicola*), and *Enterococcus* spp. + *Lactococcus lactis* ssp. *lactis (L. lactis* ssp. *lactis)*. At present, it is common to talk about non-*aureus* staphylococci (NAS) instead of CNS, as some CNS are coagulase-variable. However, due to the fact that the kit used specifically reports the CNS in the list of target DNA (https://dna-diagnostic.com/files/Product_sheet_Mastit_4_09.02.21.pdf, accessed on 10 June 2023), we prefer to be consistent with the manufacturer and use the CNS acronym. Supporting this, Lee et al. [19] concluded that CNS and NAS do not represent exactly the same group of pathogens. NAS in fact refers to both CNS and coagulase-variable staphylococci.

After thawing, milk samples were inverted for homogenization and a representative volume (0.50 mL) was extrapolated for pathogens DNA extraction according to the kit manufacturer’s instructions. The qPCR reaction was performed in the AriaMx Real-Time PCR System (Agilent Technologies Inc., Santa Clara, CA, USA) and run under the following conditions: 95 °C for 1 min for 1 cycle, and 95 °C for 5 s and 60 °C for 25 s for 40 cycles. Cycle threshold (Ct) values were reported for all samples. As recommended by the manufacturer, for all the pathogens identified in the analysis, a Ct ≤ 37 was considered a positive result (=DNA present). Samples with a Ct > 37 were considered free from pathogen DNA [8]. The assay’s protocol involved three separate multiplex real-time PCR reactions, each of which targeted four pathogens and an internal amplification control.

### 2.4. Prevalence of Pathogens

Using the qPCR outputs, pathogen frequencies were calculated. In the DHI set, only one sample per cow was randomly kept to avoid multicollinearity between observations, and for all the herd-test date levels at least 5 cows were examined. From the initial 177 herds that joined the DHI, 145 remained after this restriction. For all the dates of analysis, samples from at least 10 cows and at least 3 farms were processed in the laboratory; when present, dates where samples from fewer than 3 herds were processed were discarded. This resulted in 63 dates of analysis out of the initial 114. Apart from the β-lactamase gene (*blaZ*), which is expressed in staphylococci with penicillin resistance [20], milk samples were considered contaminated and thus discarded when presenting ≥3 isolated pathogens [21]. The final data set consisted of (i) 3239 quarter milk samples from 822 cows in 9 herds for STER and (ii) 5464 composite milk samples from the same number of cows in 145 herds for DHI (Table 1). In the two sets, the cows were comparable in terms of days in milk, parity, and milk yield and composition (Table 2). 

Based on the presence/absence of pathogen DNA in the samples, prevalence was recorded between and within (i) herds under DHI, (ii) herds under STER, and (iii) dates of analysis (DHI only). Finally, the prevalence of pathogens was calculated in samples presenting high vs. low levels of both SCC and DSCC. Separation between high and low SCC or DSCC was carried out using thresholds proposed by Schwarz et al. [23]: 200,000 cells/mL for SCC and 65% for DSCC. To assess similarity between frequencies, the Chi-square goodness of fit test was used. The null hypothesis was that the observed frequencies of the DHI matched those of the STER. The significance level was set at *p* < 0.05. Data manipulation, editing, and analysis were carried out in the software program R v. 4.1.3 [24].

## 3. Results

### 3.1. Presence of Pathogens 

In this study, prevalence of pathogens was calculated in samples collected under STER and DHI from apparently healthy cows. It is important to highlight that with the data available, it was not possible to determine the origin of the pathogen, especially with the DHI protocol. In fact, we cannot exclude the possibility that the DNA in some milk samples originated from a contaminated teat canal or skin and not from a truly infected quarter/udder. All the animals involved in the STER test (n = 822) had four functional quarters, resulting in 3288 samples (Table 1). No DNA from the twelve target pathogens was detected in 58.70% and 49.30% of the STER and DHI samples, respectively (Table 3), and the proportion of samples with one type of single-target DNA was very similar between the two sampling procedures (31.40% and 31.70%; Table 3). Contaminated samples (≥3 pathogens) represented 1.50% of STER samples and 5.80% of DHI samples. 

The prevalence of the DNA in the two sets is reported in Table 4 for all the target pathogens of the “Mastitis 4BDF” commercial kit. The prevalence of *S. agalactiae* was 3.09% and 3.13% in STER and DHI, respectively. Also, *S. aureus* and the β-lactamase gene were present at a similar frequency (*p* < 0.05) in the two sampling sets (0.99% vs. 1.45% for *S. aureus* and 4.45% vs. 5.34% for β-lactamase gene). 

Among the environmental pathogens, the prevalence of *S. dysgalactiae*, *E. coli*, and *Prothoteca* was similar between STER and DHI (Table 4). The other pathogens (i.e., *M. bovis*, *Mycoplasma* ssp., *S. uberis*, *Klebsiella*, *Enterococcus* + *L. lactis* ssp. *lactis*) had greater prevalence in DHI than STER. On the other hand, the prevalence of CNS in STER (26.55%) was almost twice that of DHI (13.87%).

### 3.2. Pathogens across Herds

In all 9 herds in the STER set, there was at least a sample (=quarter) positive for target DNA. On the other hand, only 3 herds out of the 145 of the DHI set had no samples positive for target pathogen DNA, meaning that the remaining 142 herds had at least one sample (=cow) positive for a pathogen. Under STER, all herds had at least one sample positive for DNA from *S. uberis*, *S. dysgalactiae*, CNS, and β-lactamase gene (Table 5). As regards DHI, DNA from *S. uberis*, CNS, and *Enterococcus* spp. + *L. lactis* ssp. *lactis* was detected in more than 80% of herds. In both sets, *Prothoteca* spp. was the least frequently detected pathogen, being detected in the 6.90% and 44.44% of DHI and STER herds, respectively. 

**Figure 2:**
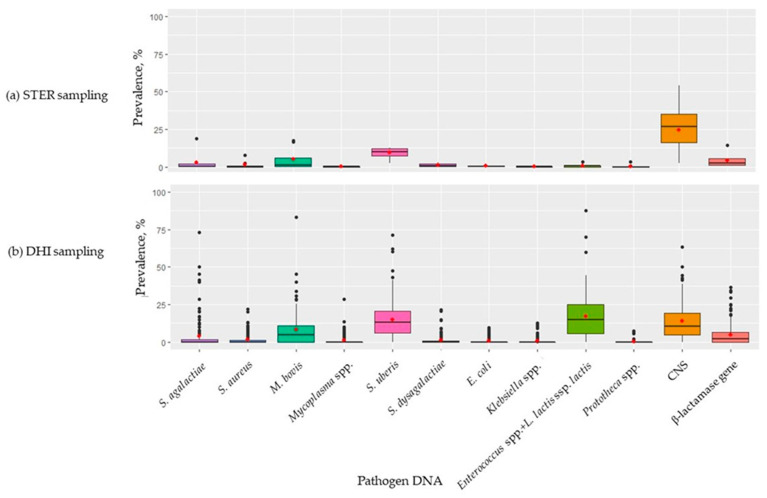
Prevalence of pathogen DNA in the (**a**) 9 herds of the sterile (STER) sampling set and (**b**) 145 herds of the dairy herd improvement (DHI) sampling set. Each black dot represents an outlier herd, whereas red dots represent the average prevalence.

Figure 2 depicts the distribution of each pathogen within herds in the two sets. In most cases, the distribution was skewed, suggesting that the majority of cows did not present the target DNA. In the herds involved in the STER test (Figure 2a), CNS was the pathogen with the greatest prevalence: the distribution was less skewed compared to the other DNA, with a median of 26.67% and maximum of 54.23%. To follow, *S. uberis* was detected with a median of 10.24% and a maximum of 12.88% within the herds. The other pathogens were detected with lower prevalence (median < 3%). DNA from *S. agalactiae*, *M. bovis*, and β-lactamase gene was present with high prevalence (18.94%, 17.34%, and 14.55%, respectively) in only one STER farm. In the DHI herds (Figure 2b), the pathogen DNA with the greatest prevalence within herds was CNS, *S. uberis*, and *Enterococcus* spp. + *L. lactis* ssp. *lactis*. The other pathogens had lower prevalence, with a median below 5%. In 4 herds, DNA from *S. agalactiae*, *M. bovis*, *S. uberis*, and *Enterococcus* spp.+ *L. lactis* ssp. *lactis* was detected in at least 70% of samples. However, it is important to highlight that these herds accounted for a limited number of samples, i.e., 15, 6, 7, and 24, respectively (Figure 2b).

**Figure 3:**
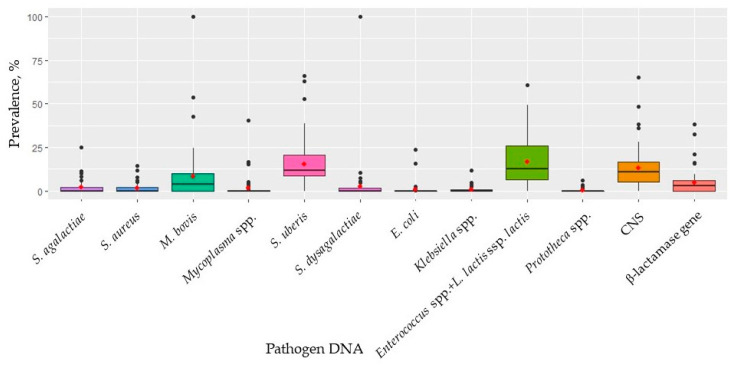
Prevalence of pathogen DNA on each date of analysis (n = 63) for the dairy herd improvement (DHI) sampling set. Each black dot represents an outlier date, whereas the red dots represent the average prevalence.

As mentioned above, samples from multiple DHI herds were often analyzed on the same day. To evaluate presence of lab contamination/carry-over, the prevalence of pathogen DNA on each date of analysis was calculated (Figure 3). On one date of analysis, all the samples processed in the laboratory (n = 22, 3 herds) had DNA from *M. bovis* and *S. dysgalactiae*. On almost all dates, however, at least one sample had DNA from *S. uberis* (n = 57), CNS (n = 56), and *Enterococcus* spp. + *L. lactis* ssp. *lactis* (n = 59).

### 3.3. Pathogens and Udder Health 

**Figure 4:**
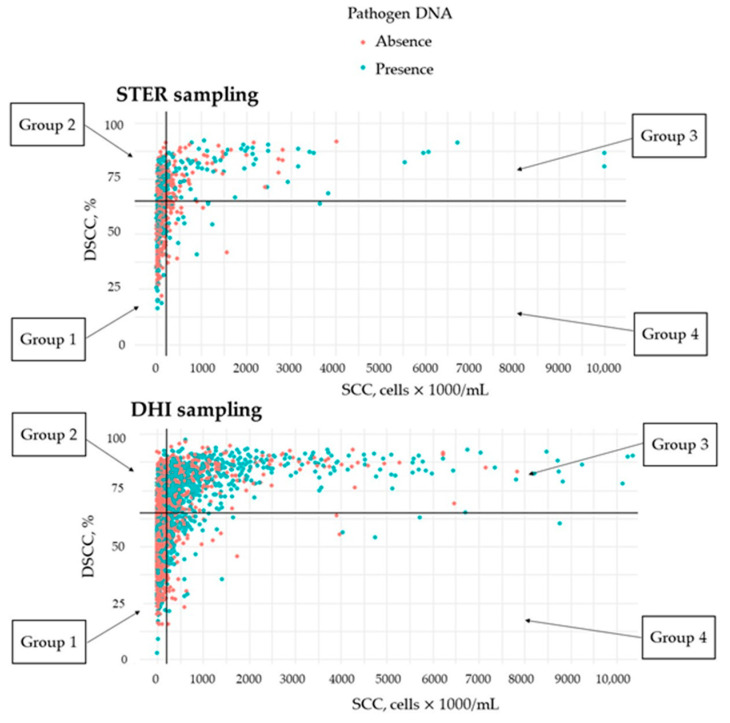
Distribution of samples with (green) and without (red) detected pathogen(s) DNA across the four udder health status groups in the sterile (STER; each sample represents the milk of a single quarter) and the dairy herd improvement sampling (DHI; each sample represents the composite milk of a cow) sets. Udder health status groups were defined in this study according to the milk level of somatic cell count (SCC) and differential SCC (DSCC). Thresholds proposed by Schwarz et al. [20] were used: group 1 (SCC < 200,000 cells/mL and DSCC < 65%), group 2 (SCC < 200,000 cells/mL and DSCC ≥ 65%), group 3 (SCC ≥ 200,000 cells/mL and DSCC ≥ 65%), and group 4 (SCC ≥ 200,000 cells/mL and DSCC < 65%).

The presence of DNA from one or more pathogens in the mammary gland secretum does not necessarily mean that mastitis is present. However, samples without pathogen DNA are more likely to be collected from cows with optimal udder health and reared in optimal hygienic conditions. By means of milk inflammation indicators, i.e., SCC and DSCC, samples were grouped as depicted in Figure 4.

The milk composition of STER samples was assessed in the composite sample of each cow, not at quarter level. Thus, the SCC and the DSCC of single quarters were not available. The 51.02%, 22.31%, 20.45%, and 6.22% of STER samples (=cows) belonged to group 1 (SCC < 200,000 cells/mL and DSCC < 65%), 2 (SCC < 200,000 cells/mL and DSCC ≥ 65%), 3 (SCC ≥ 200,000 cells/mL and DSCC ≥ 65%), and 4 (SCC ≥ 200,000 cells/mL and DSCC < 65%), respectively. Similarly, 47.67%, 26.19%, 21.36%, and 4.78% of DHI samples (=cows) fell in group 1, 2, 3, and 4, respectively (Figure 4). In group 3, where both markers of inflammation (SCC and DSCC) were high, pathogen DNA was detected more frequently than in the other groups. Samples that tested positive for target DNA accounted for 53.00% (345 out of 651) of STER and 65.38% (763 out of 1167) of DHI. In group 4, where only SCC was high, pathogen DNA was detected in 42.93% and 58.62% of STER and DHI samples, respectively. In both the sets, the group with the greatest number of samples free of pathogens was group 1, where both SCC and DSCC were low (35.28% for STER and 42.22% for DHI). Finally, the frequency of samples with pathogen DNA in group 2 was 43.10% for STER and 44.55% for DHI. 

## 4. Discussion

### 4.1. Detected Pathogens 

In this study, a considerable number of milk samples were collected from healthy cows, i.e., those without clinical mastitis nor under any antimicrobial treatments. Although two different sampling procedures were used, milk samples analyzed via qPCR belonged to cows reared in herds from the same area (Veneto region, Italy) with the same management and feeding systems, i.e., intensive farms with cows fed total-mixed ration. The prevalence of milk samples with no pathogen DNA in STER (58.70%) and DHI (49.30%) resembled the findings of Narayana et al. [21], who performed bacterial cultures on 46,900 quarter-level samples of Canadian Holsteins and reported 54.5% uninfected samples. Using qPCR for the identification of four pathogens, Timonen et al. [25] observed that pathogen DNA was absent in 62.5% of quarter milk samples collected from 133 Holsteins. Moreover, the prevalence of pathogens observed in the present paper (Table 4) agrees with the results of studies that investigated pathogen prevalence in clinically healthy cows [21,25] or in cows with detected or suspected mastitis [18,26]. 

According to Narayana et al. [21] and Vakkamäki et al. [26], the most frequently detected pathogen DNA is generally CNS. In fact, Narayana et al. [21] reported a prevalence of 30.8% for CNS, while the prevalence reported by Vakkamäki et al. [26] (46.0%) was expected to be greater compared to the present study as those authors did not include healthy cows in their study and focused exclusively on animals with detected or suspected mastitis. As anticipated, although CNS is now commonly referred to as NAS, in this study the CNS acronym was used for consistency with the “Mastitis 4BDF” kit. CNS is a group of opportunistic pathogens, naturally present on the cow’s skin, that cause clinical or subclinical inflammation only when certain conditions favor their colonization of the udder, e.g., poor milking hygiene, inadequate teat disinfection, stressed cows and/or a scarce immune response [3]. 

All pathogens detected using “Mastitis 4BDF” have been associated with the occurrence of clinical or subclinical mastitis in dairy cattle. In particular, *S. aureus* and *S. agalactiae* are the most common bacterial pathogens associated with clinical mastitis. Vakkamäki et al. [26], who detected pathogen DNA by using the PathoProof Mastitis PCR Complete-12 assay in quarter milk samples from more than 90,000 Finnish cows with mastitis in 4725 herds, reported a prevalence of 21.1% for *S. aureus*. Similar prevalence of *S. aureus* was observed by Kalmus et al. [18], who used the same qPCR assay adopted in the current study to analyze quarter milk samples of 263 Estonian cows with clinical mastitis. Prevalence was higher than that of our study on apparently healthy cows. For *S. agalactiae*, Kalmus et al. [18] reported a greater prevalence (5.3%), while Vakkamäki et al. [26] reported a very low prevalence (0.4%). Other contagious pathogens include *Mycoplasma* spp., with *M. bovis* being the most common. This contagious pathogen usually causes subclinical or mild clinical intramammary inflammation, which can progress to chronic mastitis [8]. The spread of this pathogen varies depending on the study. Timonen et al. [8], who used a qPCR assay to detect DNA from four pathogens in 522 composite milk samples, reported a prevalence of 30% for *M. bovis*. On the other hand, Vakkamäki et al. [26] reported a prevalence of 0% for both *Mycoplasma* spp. and *M. bovis*.

In agreement with Narayana et al. [21], environmental pathogens were generally more present than contagious ones. However, the presence of *S. uberis* in this study, especially in the case of DHI, was greater than that (9.0%) reported by Vakkamäki et al. [26]. *S. uberis* is recognized as one of the major environmental pathogens causing both clinical and subclinical mastitis in dairy cows [27]. *E. coli*, a gram-negative bacterium, is another significant contributor to clinical mastitis and it is often associated with acute cases and severe clinical signs [28]. While in this study, DNA from *E. coli* was detected with a prevalence lower than 1% in both sampling procedures, Kalmus et al. [18] and Vakkamäki et al. [26] observed a prevalence of 29.6% and 4.7% in milk from mastitic cows, respectively. Regarding other environmental pathogens, Vakkamäki et al. [26] reported 7.9%, 1.5%, and 0.7% prevalence for *S. dysagalactiae*, *Enterococcus* spp., and *Klebsiella* spp., respectively. Kalmus et al. [18] observed *S. dysagalactiae* in 14.6% of the samples, *Klebsiella* spp. in 0.9% and *Enterococcus* spp. in 1.7%. The prevalence of *Klebsiella* spp. is in line with the prevalence observed in the STER and DHI sets, while for *Enterococcus* spp., only the STER prevalence was similar to that reported by Kalmus et al. [18]. It is likely that the DHI prevalence was greater due to contamination of the sample during collection since bovine faeces are a source of this microorganism [29]. The prevalence of *S. dysagalactiae* in this study was lower because apparently healthy cows were sampled. Finally, DNA from *Prototheca* spp. was detected with a rate lower than 1% in both this study and Vakkamäki et al. [26]. *Prototheca* spp. are algae organisms that can invade the udder tissue, leading to inflammation and are associated with clinical and subclinical mastitis [30]. 

### 4.2. Milk Pathogens Detection via qPCR

The identification of etiological agents is relevant in dairy cattle farming in order to choose targeted solutions aimed at reducing or eradicating specific pathogens responsible for intramammary infection. Although rather costly, detection of pathogens via qPCR in bulk or individual cow milk allows for farm-level screening and handling of clinical or subclinical mastitis cases. In field conditions, rapid analysis on a large number of samples for an immediate response is fundamental for decision-making. Therefore, the use of the bacteria culture tests is not suitable on a routine basis, mainly due to the long analysis time and to the fact that samples with no preservatives are required. This opens the debate on the possibility of using qPCR mastitis diagnostic tests, which are faster, more objective, and applicable to frozen samples or samples containing preservatives.

Comparison of qPCR with the bacterial culture has already been performed in cow milk for different pathogens [11,31,32]. The performance of the two methods was compared via a latent class analysis. Some authors used the same qPCR assay as the present study [31,32] while others used different kits such as the PathoProof Mastitis qPCR assay [11]. Svennesen et al. [32] investigated the mastitis diagnostic accuracy of both qPCR and bacteria cultures for two pathogens, *S. agalactiae* and *S. aureus*, in quarter samples collected using aseptic technique. In that study, the accuracy was higher for qPCR compared to the bacterial culture with sensitivity and specificity of 0.97 and 0.96 for *S. agalactiae*, and 0.94 and 0.98 for *S. aureus*, respectively. Nyman et al. [11] remarked that using qPCR in non-aseptically collected composite milk samples increased the sensitivity for *S. aureus*, *S. agalactiae*, *S. uberis*, and CNS, while for other pathogens the specificity remained stable or reduced compared to bacterial culture of aseptically collected quarter milks due to carry-over.

Certainly, qPCR technologies for the identification of bovine mastitis pathogens on non-aseptically collected composite milk samples (DHI sampling) have some limitations such as the higher risk of false positives due to carry-over during and after sampling. In farm settings, for example, the milking unit can contain residual milk from the previous cow, and the transfer of droplets of contaminated milk is often not negligible [33]. At the laboratory level, analysis via infrared spectroscopy with instruments like the Combifoss^TM^ 7 is the core of the DHI test as it determines phenotypes adopted by national organizations for performance monitoring and genetic selection for factors such as content of fat, protein, lactose, and urea, and SCC. If samples are analyzed through qPCR after this step, the risk of having biased results due to carry-over increases. 

However, the choice of Ct value cut-off when interpreting PCR results affects the amount of DNA which is reported as a positive result. Consequently, Ct value cut-off can impact the number of false positives and the degree of carry-over. According to Mahmood et al. [12,13], selecting a higher Ct value cut-off increases sensitivity, meaning it detects more positive cases, but it increases also the degree of carry-over, which refers to the contamination of samples leading to false positives. On the other hand, choosing a lower Ct value cut-off, which represents heavily or truly infected cows, decreases the number of both true positive (correctly identified infected cows) and false positive cases (cows incorrectly identified as infected). In summary, the choice of Ct value cut-off in PCR interpretation is a trade-off between sensitivity and specificity. A higher cut-off increases sensitivity but also the risk of false positives due to carry-over, while a lower cut-off decreases both true and false positive cases [12,13]. 

In this study, the two possible forms of carry-over were checked by investigating the prevalence of pathogens between and within (i) herds and (ii) dates of analysis. If there was a high prevalence of pathogens in a herd (Figure 2b) or date of analysis (Figure 3), this was interpreted as occurrence of crossover during sampling or during infrared analysis in the laboratory. Overall, although the results suggest that contamination or carry-over took place, it is important to bear in mind that on some dates of analysis or in some herds of the DHI set, the number of analyzed samples was very low. In fact, the frequencies identified as extreme (outliers) were those of small herds or days where only 6 samples were analyzed. As an example, DNA from *S. dysgalactiae* and *M. bovis* was detected in all milks from a single date, suggesting that carry-over most likely occurred in the laboratory during/after the infrared analysis, prior to starting qPCR analysis. As regards the prevalence of *S. uberis* in the DHI set (Table 4, Figure 2b), cross-contamination may have occurred at the farm level. *S. uberis* is a ubiquitous microorganism usually isolated from the environment, and barn bedding material represents the main site of infection and contamination [34]. Considering its prevalence in Table 4 and Figure 2b, *Enterococcus* spp. cross-contamination most likely also occurred, probably during sampling since bovine feces are a source of this microorganisms [29]. *L. lactis*, which is commonly detected on animal skin [35] is included on the Qualified Presumption of Safety list of the European Food Safety Authority (EFSA, Parma, Italy); even though generally recognized as safe, it can cause mastitis in dairy cows if conditions are favorable [36]. 

The prevalence of DNA from *S. aureus*, *S. agalactiae*, *S. dysgalactiae*, *E. coli*, *Prototheca*, and the β-lactamase gene was similar between the STER and DHI sets (*p* > 0.05). A specific note is needed for CNS whose DNA was detected in milk samples of DHI (Table 4, Figure 2b). The prevalence observed can be considered reliable even if it was lower than that of the STER set; it is reasonable to think that the difference can be attributed to a dilution of the DNA in the composite samples compared to quarter-level samples. In other words, in the composite milk (pool of all quarters) the DNA concentration of CNS was under the detection limit of the qPCR, even though qPCR is very sensitive [37]. Another potential reason could be the presence of false positives. The CNS are commonly found on the teat skin [3] and their presence in the sample is reasonable if the sampling was made with manipulation of teats like in the STER. 

Finally, it is important to highlight that the kits used in the present study allowed detection of eleven pathogens solely, thus authors cannot exclude that some samples had DNA from non-target pathogens. 

### 4.3. Overview of Udder Health

Milk free of pathogen DNA is expected to be harvested from apparently healthy cows free of any infections. When present in milk, the surrounding environment, or in the mammary gland skin, some pathogens increase the exposure of cows and the probability of clinical or subclinical forms of mastitis. The qPCR can provide a broader picture of the udder health status of cows, especially if used in conjunction with SCC and farm data. The DSCC has been introduced in recent years as a promising trait to improve the accuracy of mastitis detection [11], but still the realized boost has yet to be demonstrated on a large scale, and there are concerns regarding the real usefulness and the interpretation of this trait. According to Leitner et al. [38], DSCC varies along a temporal axis in relation to the inflammation event, that is, before, during, and after. In particular, authors reported that DSCC is very high in early stages of infection, while in chronic cases the proportion of macrophages increases and the DSCC tends to decrease [38,39].

The distribution of milk samples across the four udder health status groups were similar between the STER and DHI sets (Figure 3). In fact, most of the samples belonged to group 1 in both sets where SCC and DSCC were low, while group 4 (high SCC and low DSCC) was the least represented group. Considering how samples with and without detected pathogen DNA were distributed across the four udder health status groups, it can be argued that the presence of pathogens is not always indicative of high SCC and/or DSCC. This corroborates the idea that certain pathogens’ DNA in milk is likely to originate from the milk canal, teat skin, or other contamination during sampling (especially for DHI protocol) and not from a truly infected quarter/udder. On the other hand, the presence of certain pathogens’ DNA does not necessarily translate into an increase of markers of inflammation (SCC and DSCC), probably due to scarce immune response of specific cows. The kit used in the present study has been developed for twelve targets and high SCC and DSCC in milk could be thereby related to the presence of other pathogens, here not detectable (e.g., *Corynebacterium* spp., *Pseudomonas* spp.). There are a large number of bacteria, algae, and fungi that cause mastitis in cows [40] and could not be detected with the “Mastitis 4BDF” kit. However, at the same time, it is worth mentioning that in this study, cows with clinical mastitis were excluded and that both SCC and DSCC must be considered indicators of udder health, i.e., their correlation with mastitis can be moderate or strong, but far from unity, and their variability is affected by management and cow-related factors (i.e., breed, stage of lactation, parity, and season of sampling). Samples originating from cows with quarters with pathogen DNA could show low SCC and DSCC (group 1, Figure 3). In fact, if a cow has only a single quarter with pathogen DNA, the increase of SCC and DSCC in the composite milk sample is masked due to dilution of the cells. Moreover, the increase of SCC and DSCC depends on the immune response which can be pathogen-specific [41]. Schwarz et al. [42] observed that, if mastitis is caused by major pathogens such as *S. aureus*, *E. coli*, *S. uberis*, *S. dysgalactiae*, the combination of DSCC and SCC help to improve sensibility for mastitis identification because DSCC increases drastically [42].

Although validation of the protocol using “Mastitis 4BDF” kit on composite milk samples with large data sets is still needed, in perspective, whether combined with SCC and DSCC, qPCR data can provide valuable information regarding udder latent infections or subclinical inflammations. A better knowledge on pathogen-specific pattern of SCC and DSCC can be useful for the definition of novel indicator traits to be exploited for breeding purposes, e.g., to select for pathogens resistance [6].

## 5. Conclusions

Results suggest that in northern Italy, the most common DNA in the udder of healthy cows, regardless of the sampling protocol used (sterile sampling carried out at quarter-level or conventional sampling in non-aseptic conditions), is that of CNS, followed by *S. uberis*. Among contagious pathogen DNA, *M. bovis* was the most present, followed by *S. agalactiae*. Findings indicate that the prevalence of DNA from *S. aureus*, *S. agalactiae*, β-lactamase gene, *E. coli*, and *Prothoteca* spp. calculated within the two sampling procedures is similar. In addition, regardless of the sampling protocol adopted, when udder health-related traits (i.e., SCC and DSCC) were high, pathogens were detected more frequently than in the other cases. Rapid pathogen detection tests such as qPCR assay may represent a potential screening tool of interest for dairy farmers. For instance, whenever validated with a “gold standard” test such as bacteriological cultures, qPCR can be used to evaluate the presence of some target pathogens and the individual udder health, and for decision-making such as selective dry cow therapy. It still remains unclear if qPCR results obtained on non-aseptically collected composite milk samples—DHI sampling—significantly differ from qPCR results obtained using sterile milk. If not, qPCR could be potentially used in individual samples routinely collected on a monthly basis for official milk analysis, especially for cows close to the dry period. Future studies in this direction should explore this opportunity for dairy stakeholders. 

## Figures and Tables

**Table 1:** Number of milk samples available for the sterile (STER, quarter-level samples) and the dairy herd improvement (DHI, composite samples) sampling sets before and after the restrictions.

Item	STER Sampling	DHI Sampling
Initial database	3288 (822 cows)	9471 (5999 cows)
Edited database	3239 (822 cows)	5464 (5464 cows)
Holstein Friesian	1589	4876
Simmental	625	252
Rendena	559	271
Jersey	466	65

**Table 2:** Mean (standard deviation) of parity, days in milk, milk yield, and milk composition of samples collected through the sterile (STER) and the dairy herd improvement (DHI) sampling.

Item	STER Sampling	DHI Sampling
Parity, n	2.57 (1.77)	2.58 (1.47)
Days in milk, d	226 (138)	205 (155)
Milk yield, kg/d	26.41 (9.74)	30.40 (11.53)
SCS ^1^	2.95 (2.01)	2.88 (1.99)
Differential SCC, %	61.89 (15.16)	62.59 (15.78)
Fat, %	3.91 (0.83)	4.31 (0.97)
Protein, %	3.58 (0.44)	3.55 (0.44)
Lactose, %	4.72 (0.29)	4.76 (0.24)

^1^ SCS = somatic cell score, calculated as log-transformation of somatic cell count (SCC) [22].

**Table 3:** Prevalence of milk samples with 0, 1, 2, or more detected pathogens in the sterile ^1^ and the dairy herd improvement (DHI) ^2^ sampling sets.

Detected Pathogens	STER Sampling		DHI Sampling	
	n	%	n	%
0	1929	58.70	2858	49.30
1	1034	31.40	1839	31.70
2	276	8.40	767	13.20
≥3 ^3^	49	1.50	335	5.80

^1^ STER; each sample represents the milk of a single quarter. ^2^ DHI; each sample represents the composite milk of one cow. ^3^ Excluded from the frequency calculation due to contamination [21].

**Table 4:** Prevalence (%) of pathogen DNA detected in the sterile ^1^ (n = 3239) and the dairy herd improvement (DHI ^2^; n = 5464) sampling sets with Chi-square test results and significance (*p*).

Pathogen DNA	Prevalence (%)		Chi-Square	*p*
	STER Sampling	DHI Sampling		
Contagious				
*S. agalactiae*	3.09	3.13	0	0.964
*S. aureus*	0.99	1.45	3.03	0.082
*M. bovis*	5.19	8.47	32.18	<0.001
*Mycoplasma* spp.	0.19	0.93	16.36	<0.001
Environmental				
*S. uberis*	9.60	16.53	80.66	<0.001
*S. dysagalactiae*	1.02	1.41	2.18	0.140
*E. coli*	0.48	0.68	0.85	0.358
*Klebsiella* spp.	0.28	0.77	7.59	0.006
*Enterococcus + L. lactis* ssp. *lactis*	1.02	14.17	417.77	<0.001
*Prototheca* spp.	0.56	0.33	2.53	0.112
Opportunistic				
CNS	26.55	13.87	215.15	<0.001
Other				
β-lactamase gene ^3^	4.45	5.34	3.26	0.071

^1^ STER; each sample represents the milk of a single quarter. ^2^ DHI; each sample represents the composite milk of a cow. ^3^ Penicillin resistance β-lactamase gene from *staphylococci*.

**Table 5:** Percentage (%) of herds positive to a specific pathogen in the sterile ^1^ and the dairy herd improvement (DHI) ^2^ sampling sets (at least one milk sample with DNA detected).

Pathogen DNA	STER Sampling	DHI Sampling
Contagious		
*S. agalactiae*	66.67	26.21
*S. aureus*	77.78	27.59
*M. bovis*	88.89	64.14
*Mycoplasma* spp.	55.56	20.69
Environmental		
*S. uberis*	100	83.45
*S. dysagalactiae*	100	26.21
*E. coli*	88.89	13.79
*Klebsiella* spp.	66.67	17.24
*Enterococcus + L. lactis* ssp. *lactis*	66.67	86.21
*Prototheca* spp.	44.44	6.90
Opportunistic		
CNS	100	83.45
Other		
β-lactamase gene ^3^	100	53.86

^1^ STER; each sample represents the milk of a single quarter. ^2^ DHI; each sample represents the composite milk of a cow. ^3^ Penicillin resistance β-lactamase gene from *staphylococci*.

## Data Availability

The datasets generated during and/or analyzed during the current study are available from the corresponding author on reasonable request.
